# Bim and Mcl-1 exert key roles in regulating JAK2^V617F ^cell survival

**DOI:** 10.1186/1471-2407-11-24

**Published:** 2011-01-19

**Authors:** Joëlle Rubert, Zhiyan Qian, Rita Andraos, Daniel A Guthy, Thomas Radimerski

**Affiliations:** 1 Disease Area Oncology, Novartis Institutes for BioMedical Research, Basel, Switzerland

## Abstract

**Background:**

The JAK2^V617F ^mutation plays a major role in the pathogenesis of myeloproliferative neoplasms and is found in the vast majority of patients suffering from polycythemia vera and in roughly every second patient suffering from essential thrombocythemia or from primary myelofibrosis. The V617F mutation is thought to provide hematopoietic stem cells and myeloid progenitors with a survival and proliferation advantage. It has previously been shown that activated JAK2 promotes cell survival by upregulating the anti-apoptotic STAT5 target gene Bcl-xL. In this study, we have investigated the role of additional apoptotic players, the pro-apoptotic protein Bim as well as the anti-apoptotic protein Mcl-1.

**Methods:**

Pharmacological inhibition of JAK2/STAT5 signaling in JAK2^V617F ^mutant SET-2 and MB-02 cells was used to study effects on signaling, cell proliferation and apoptosis by Western blot analysis, WST-1 proliferation assays and flow cytometry. Cells were transfected with siRNA oligos to deplete candidate pro- and anti-apoptotic proteins. Co-immunoprecipitation assays were performed to assess the impact of JAK2 inhibition on complexes of pro- and anti-apoptotic proteins.

**Results:**

Treatment of JAK2^V617F ^mutant cell lines with a JAK2 inhibitor was found to trigger Bim activation. Furthermore, Bim depletion by RNAi suppressed JAK2 inhibitor-induced cell death. Bim activation following JAK2 inhibition led to enhanced sequestration of Mcl-1, besides Bcl-xL. Importantly, Mcl-1 depletion by RNAi was sufficient to compromise JAK2^V617F ^mutant cell viability and sensitized the cells to JAK2 inhibition.

**Conclusions:**

We conclude that Bim and Mcl-1 have key opposing roles in regulating JAK2^V617F ^cell survival and propose that inactivation of aberrant JAK2 signaling leads to changes in Bim complexes that trigger cell death. Thus, further preclinical evaluation of combinations of JAK2 inhibitors with Bcl-2 family antagonists that also tackle Mcl-1, besides Bcl-xL, is warranted to assess the therapeutic potential for the treatment of chronic myeloproliferative neoplasms.

## Background

The somatic activating JAK2^V617F ^mutation is found in nearly every patient with the chronic myeloproliferative neoplasm (cMPN) polycythemia vera (PV) and roughly half of those patients affected by essential thrombocythemia (ET) and primary myelofibrosis (PMF) [1]. At the molecular level, it is thought that the V617F mutation in the JAK2 pseudokinase alleviates some of the negative regulation that this domain normally elicits on the kinase domain [2], allowing for increased kinase autoactivation [3]. Clinical trials with JAK inhibitors in primary myelofibrosis patients are underway and have shown rapid suppression of splenomegaly and improvement of constitutional symptoms [4]. However, up to now effects on mutant allele burden have been modest and bone marrow fibrosis appears to persist [5], warranting continued pre-clinical and clinical research in order to improve therapeutic outcome of JAK inhibitors in cMPNs. Mutant JAK2^V617F^, which arises at the level of the hematopoietic stem cell [6], likely provides progenitor cells with both a proliferation and a survival advantage [7]. Hence, a potential avenue for enhanced JAK2^V617F ^cell killing by JAK2 inhibitors may lie in simultaneous perturbation of survival mechanisms. Importantly, several studies have found that the anti-apoptotic Bcl-2 family member Bcl-xL plays a role in PV erythroblast survival [8,9]. Along these lines, Bcl-xL depletion induced apoptosis in JAK2^V617F ^mutant cells and the BH3 (Bcl-2-homology domain 3)-mimetic ABT-737 was shown to preferentially kill JAK2^V617F ^mutant PV erythroid precursors as compared to healthy subject erythroblasts [9,10]. The BH3-only pro-apoptotic protein Bad has been implicated in regulating JAK2^V617F ^mutant cell survival [10] and engages anti-apoptotic Bcl-2, Bcl-xL and Bcl-w, but not Mcl-1 [11]. Mcl-1 protein is normally short-lived due to rapid proteasome-mediated destruction but contributes to resistance to cell-death stimuli if its levels are elevated [12,13].

In this study we focused on elucidating potential roles of pro-apoptotic Bim and anti-apoptotic Mcl-1 in regulating JAK2^V617F ^mutant cell survival. In contrast to Bad, Bim can engage all Bcl-2 pro-survival family members, including Mcl-1 [11]. Both Bim and Mcl-1 were readily detectable in JAK2^V617F ^mutant cell lines and co-immunoprecipitated. JAK2 inhibition led to changes in Bim-EL Ser69 phosphorylation, along with a drop in total Mcl-1 levels and concomitant induction of programmed cell death. In support of a key role in regulating JAK2^V617F ^cell survival, Mcl-1 depletion by RNAi was found to severely compromise cell viability and sensitized cells to JAK2 inhibition. Taken together, we show that Mcl-1 appears to be critical for JAK2^V617F ^mutant cell survival, and corroborate that cell death induced by JAK2 inhibition requires Bim activation. Our findings suggest that combinations of JAK2 inhibitors with Bcl-2 family antagonists that tackle both Bcl-xL and Mcl-1 merit further preclinical evaluation of the therapeutic potential for the treatment of cMPNs.

## Methods

### Compounds and formulations

NVP-BSK805 (free base) was synthesized internally [14], 10 mM stock solutions were prepared in dimethyl sulfoxide (DMSO) and aliquots were stored at -20°C until use. The ethyl-ester of the pan-caspase inhibitor Z-VAD-FMK was synthesized internally. UO126 (# 1144, Tocris Bioscience, Ellisville, MO, USA) was prepared as a 10 mM stock solution in DMSO and stored at -20°C until use. Obatoclax mesylate (# S1057, Selleck Chemicals, Houston, TX, USA) was prepared as a 10 mM stock solution in DMSO and stored at -20°C until use.

### Cell culture

SET-2 cells (generously provided by Prof. Hans Drexler, DSMZ, Braunschweig, Germany) were cultured in standard RPMI medium supplemented with 10% of fetal calf serum (FCS), 2 mM L-glutamine and 1% (v/v) penicillin/streptomycin. MB-02 cells (generously provided by Prof. Doris Morgan, Drexel University, Philadelphia, PA, USA) were grown in RPMI medium as described above, supplemented with 10 ng/ml recombinant human GM-CSF (granulocyte-macrophage colony-stimulating factor), 10 ng/ml recombinant human SCF (stem cell factor) and 10 mM sodium pyruvate. TF-1 cells (American Type Culture Collection) were cultured in RPMI medium, supplemented with 20% of fetal bovine serum, 1 mM L-glutamine, 5 g/l sodium bicarbonate, 10 mM HEPES, 1 mM sodium pyruvate, 4.5 g/l D-glucose, 1% (v/v) penicillin/streptomycin and 2 ng/ml GM-CSF.

### RNA interference

The following stealth™ RNAi oligonucleotides (Invitrogen, Carlsbad, CA, USA) were used; BAD: duplex 1 5'-GCUCCGGAGGAUGAGUGACGAGUUU-3', duplex 2 5'-GGACUCCUUUAAGAAGGGACUUCCU-3' and duplex 3 5'-UCUUCCAGUCCUGGUGGGAUCGGAA-3'; Bim: duplex 1 5'-UGAGUGUGACCGAGAAGG UAGACAA-3', duplex 2 5'-CAUGAGUUGUGACAAAUCAACACAA-3' and duplex 3 5'-CACGAAUGGUUAUCUUACGACUGUU-3'; Mcl-1: duplex 1 5'-GAAAGUAUCACAGACGUUCUCGUAA-3', duplex 2 5'-CGGGACUGGCUAGUUAAACAAAGAG-3' and duplex 3 5'-GGUUUGUGGAGUUCUUCCAUGUAGA-3', and a non-targeting control stealth™ RNAi oligo 5'-GAUGAAGGGAGGGUGUACCAACUUA-3'. Cells were transfected with RNAi oligonucleotides using Nucleofactor™ Solution V (Amaxa GmbH, Cologne, Germany) and the Amaxa system according to the instructions of the manufacturer.

### Real-Time Quantitative PCR

*Mcl-1 *mRNA levels were determined by real-time quantitative PCR using the Applied Biosystems Taqman Gene Expression kit (# Hs03043899_m1, Applied Biosystems, Carlsbad, CA, USA). Total RNA from cells was isolated with the RNeasy Mini Kit (# 74104, Qiagen, Hilden, Germany), accompanied by an on-column DNase (# 79254, Qiagen) digestion. Expression levels of the housekeeping gene *GAPDH *(# 4310884E, Applied Biosystems) were also measured as an endogenous normalization control. *Mcl-1 *and *GAPDH *signals were measured with FAM (6-carboxy-fluorescein) and VIC fluorescent reporter dye labeling, respectively. The volume of each reaction was 10 μl per well (384-well plate), which consisted of 5 μl 2 × reaction buffer and 0.05 μl 200 × Euroscript RT (reverse transcriptase) enzyme and RNase inhibitor mix from the one-step RT-qPCR MasterMix Plus (# RT-QPRT-032X, Eurogentec, Seraing, Belgium), 0.5 μl 20 × Taqman Gene Expression mix together with 2 μl of 50 ng RNA as amplification template. The ROX reference dye was present in the RT-qPCR reaction buffer. RT-qPCR was carried out on the ABI 7900HT Fast Real-Time PCR system (Applied Biosystems, SDS2.3 software). The reaction mixtures were incubated at 48°C for 30 minutes, during which the reverse transcription took place, 95°C for 10 minutes to activate HotGoldStar DNA polymerase (Eurogentec), followed by 40 cycles at 95°C for 15 seconds and 60°C for 1 minute. Samples were measured in triplicate. Cycle threshold (Ct) values were used to determine the relative amounts of *Mcl-1 *and *GAPDH *mRNA levels in the samples. 2^-Ct ^*Mcl-1 *values were computed and normalized to mean 2^-Ct ^*GAPDH *values. *Mcl-1 *mRNA levels were depicted as fold change compared to DMSO vehicle control by dividing normalized 2^-Ct ^values of compound treated samples by those of vehicle treated samples.

### Western blotting

Cells were extracted in lysis buffer (50 mM HEPES pH 7.4, 150 mM NaCl, 25 mM β-glycerophosphate, 25 mM NaF, 5 mM EGTA, 1 mM EDTA, 15 mM pyrophosphate PPI, supplemented freshly with 1% Nonidet P-40, 1 x protease inhibitor cocktail (Complete Mini, Roche), 1 mM DTT, 0.2 mM sodium-vanadate and 1 mM PMSF) by passing through a 1 ml syringe connected to a 23-gauge needle. Cell debris were pelleted by centrifugation. Typically, 20 μg of protein lysates were resolved by NuPAGE Novex 4-12% Bis-Tris Midi Gels (Invitrogen, Carlsbad, CA, USA) and transferred to PVDF membranes by semi-dry blotting. The following antibodies were used to probe blots: Anti-cleaved caspase 3 (# 9664), 7 (# 9491), 8 (# 9496), 9 (# 9501), Bad (#9292), Bak (# 3814), Bax (# 2772), Bcl-xL (# 2762), Bim (# 2933), phospho-Bim (Ser55 (Homo sapiens: Ser59) (# 4550), phospho-Bim (Ser69) (# 4581), ERK1/2 (# 9102), phospho-ERK1/2 (Thr201/Tyr204) (9101), Mcl-1 (# 4572), PARP (# 9542), phospho-STAT5 (# 9359) and phospho-tyrosine (# 9411) were from Cell Signaling Technology (Beverly, MA, USA). Anti-Bim (# 202000) from Calbiochem (San Diego, CA, USA) was also used. The STAT5 antibody (# sc-835) was from Santa Cruz Biotechnology (Santa Cruz, CA, USA). The β-tubulin (# T4026) and Mcl-1 (# AAP-240) antibodies were from Sigma (St. Louis, MO, USA) and Assay Designs (Ann Arbor, MI, USA), respectively. Antibodies were typically incubated overnight at 4°C followed by washes and incubation with the corresponding HRP-conjugated secondary antibodies. Immunoreactive bands were revealed with enhanced chemiluminescence reagents.

### Immunoprecipitation and co-immunoprecipitation assays

Cells were extracted either in CHAPS lysis buffer [15] or in Triton/glycerol ("TG") lysis buffer [16] (the latter for Bcl-xL/Bax co-immunoprecipitation studies), lysates were kept on ice and protein content was determined by Bradford assay. Immediately thereafter, typically 500 μg total protein input were subject to immunoprecipitation using the following antibodies: Anti-Bim (# 2933), anti-Bcl-xL (# 2762) and anti-Bax (# 2772) were from Cell Signaling Technology (Beverly, MA, USA), anti-Mcl-1 (# 559027) from BD Biosciences (Franklin Lakes, NJ, USA). Co-immunoprecipitation assays were carried out using 1.5 ml Eppendorf protein LoBind Tubes (# 0030 108.116, Eppendorf, Hamburg, Germany). Bound fractions were released by heating at 70°C for 10 minutes in 20 μl NuPAGE LDS sample buffer. The supernatant containing the bound fraction was resolved by gradient gel electrophoresis and transferred to PVDF membranes for Western blot analysis as described above.

### Proliferation assays

Anti-proliferative activity of the JAK2 inhibitor NVP-BSK805 was determined by incubating SET-2 cells or MB-02 cells with an 8 point concentration range of compound and cell proliferation relative to DMSO treated cells was measured (typically after 72 hours for SET-2 cells and after 96 hours for MB-02 cells, unless specified otherwise) using the colorimetric WST-1 (Roche Diagnostics GmbH, Penzberg, Germany) cell viability readout. Of each triplicate treatment the mean was calculated and these data were plotted in XLfit 4 (XLfit 4, ID Business Solutions Ltd, Guildford, Surrey, UK) to determine the respective half-maximal growth-inhibitory concentration (GI_50_) values.

### Flow cytometry

Cultured cells were collected after treatments, washed once with PBS and resuspended in propidium iodide buffer (1 mM sodium citrate (pH 4.0), 1.5 mM NaCl, 5 mM EDTA, 5 mM EGTA, 0.1% NP40, 4 μg of propidium iodide/ml and 80 μg/ml of RNaseA in PBS). After 30 minutes of incubation in the dark on ice, cellular DNA content was measured with a BD FACSCalibur flow cytometer (BD Biosciences, San Jose, CA, USA). For detection of activated Bak, cells were washed in PBS and then fixed at RT for 5 minutes in 0.25% paraformaldehyde (diluted in PBS). After washing twice with PBS the cell pellet was resuspended in 200 μl PBS containing 0.1% digitonin (# D141, Sigma) and then 10 μl mouse anti-Bak antibody (# AM03 (Ab-1), Calbiochem) were added followed by incubation on ice for 30 minutes. After washing twice with PBS the cell pellet was resuspended in 100 μl PBS and incubated at room temperature in the dark for 40 minutes with 5 μl fluorescein isothiocyanate (FITC)-conjugated anti-mouse antibody (# sc-2010, Santa Cruz) followed by two washes with PBS and flow cytometry analysis. The shifts in fluorescent channel 1-height (FL1-H) fluorescence intensity compared to DMSO vehicle controls were quantified and represented as fold change over DMSO control.

### Statistical analysis

The *t*-test was conducted to determine statistical significance between two groups. The significance level was set at p < 0.05. Statistical analysis was performed using SigmaPlot v11.0 (Systat Software Inc, Chicago, IL, USA).

## Results

### NVP-BSK805 JAK2 inhibitor triggered cell death requires activation of caspase cascades and is overcome by caspase inhibition

**Figure 1:**
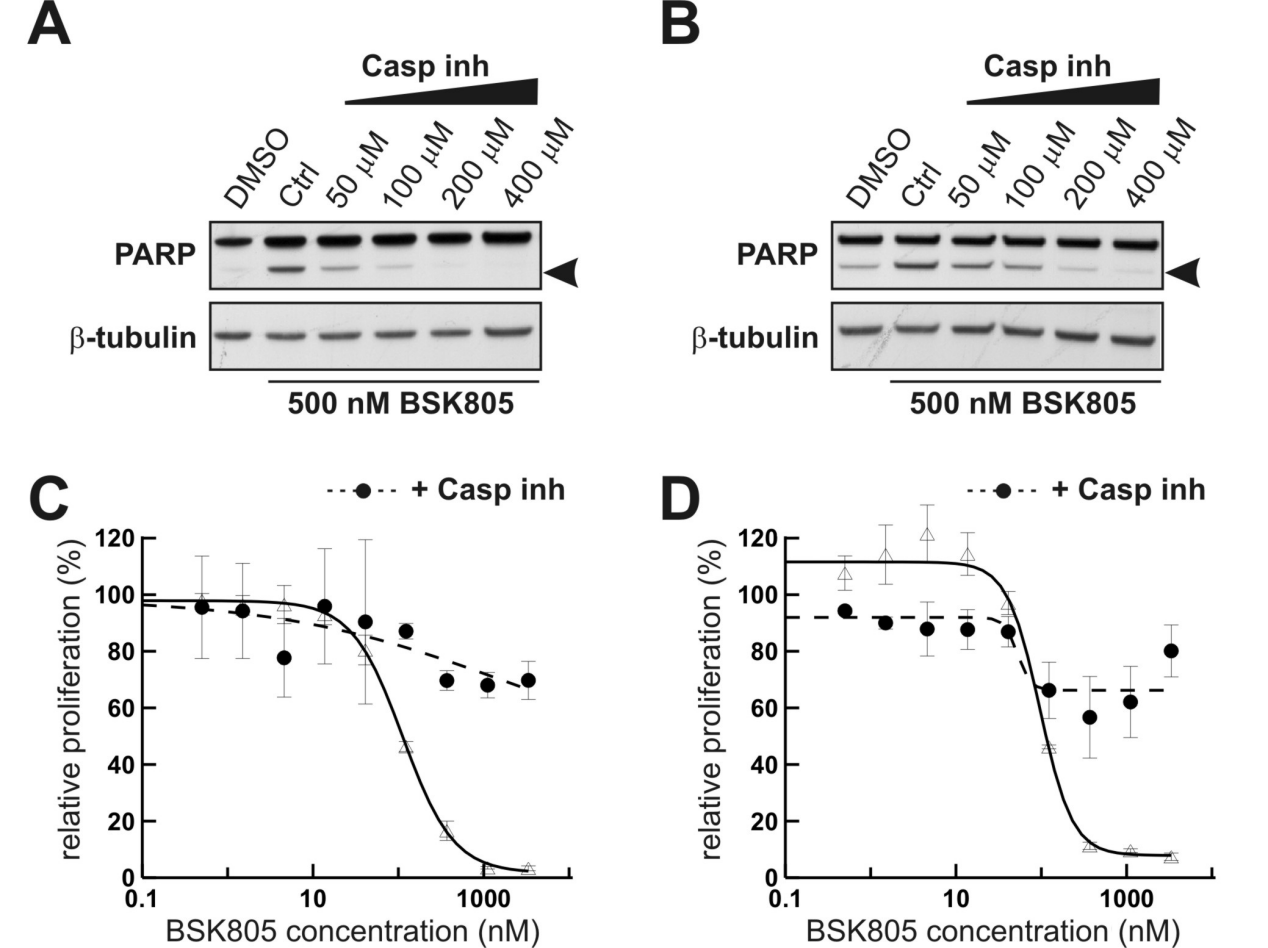
**Caspase inhibition rescues JAK2^V617F ^mutant cells from the apoptotic and anti-proliferative effects of the JAK2 inhibitor NVP-BSK805**. SET-2 (**A**) and MB-02 (**B**) cells were pretreated for 1 hour with increasing concentrations of a pan-caspase inhibitor, followed by treatment for 24 hours with 500 nM NVP-BSK805. PARP cleavage (cleaved PARP is depicted by arrowheads) was assessed by Western blot analysis. β-tubulin was probed for as a loading control. Controls consisted of drug vehicle (DMSO) treated cells (baseline) and NVP-BSK805 treatment in the absence of the pan-caspase inhibitor. Proliferation assays with increasing concentrations of NVP-BSK805 in SET-2 (**C**) and MB-02 (**D**) cells were carried out in the absence (empty triangles/solid line) or presence (filled circles/stippled line) of 200 μM pan-caspase inhibitor.

We have previously shown that the JAK2 inhibitor tool compound NVP-BSK805 blunts constitutive STAT5 phosphorylation in JAK2^V617F ^mutant cell lines, reduces Bcl-xL levels and blocks cell proliferation with concomitant induction of cell death [17]. To corroborate that NVP-BSK805 induces programmed cell death in JAK2^V617F ^mutant cell lines, we assessed if apoptosis would be overcome by pharmacological inhibition of caspases. To this end we used SET-2 and MB-02 cells, which bear mutated JAK2^V617F ^and have been derived from leukemia patients with a previous history of essential thrombocythemia and myelofibrosis, respectively [18,19]. SET-2 and MB-02 cells were pretreated for 1 hour with increasing concentrations of a pan-caspase inhibitor, followed by treatment with 0.5 μM NVP-BSK805 for 24 hours. In both cell lines the caspase inhibitor elicited a concentration-dependent suppression of JAK2 inhibitor-triggered PARP cleavage (Figure 1A and 1B). A concentration of 200 μM of the caspase inhibitor was found to completely counteract PARP-cleavage as a result of JAK2 inhibition in both cell lines. Both SET-2 and MB-02 cells respond sensitively to JAK2 inhibition by NVP-BSK805 in cell proliferation assays over 72 and 96 hours, respectively [17], and this anti-proliferative response is blunted by caspase inhibition (Figure 1C and 1D).

**Figure 2:**
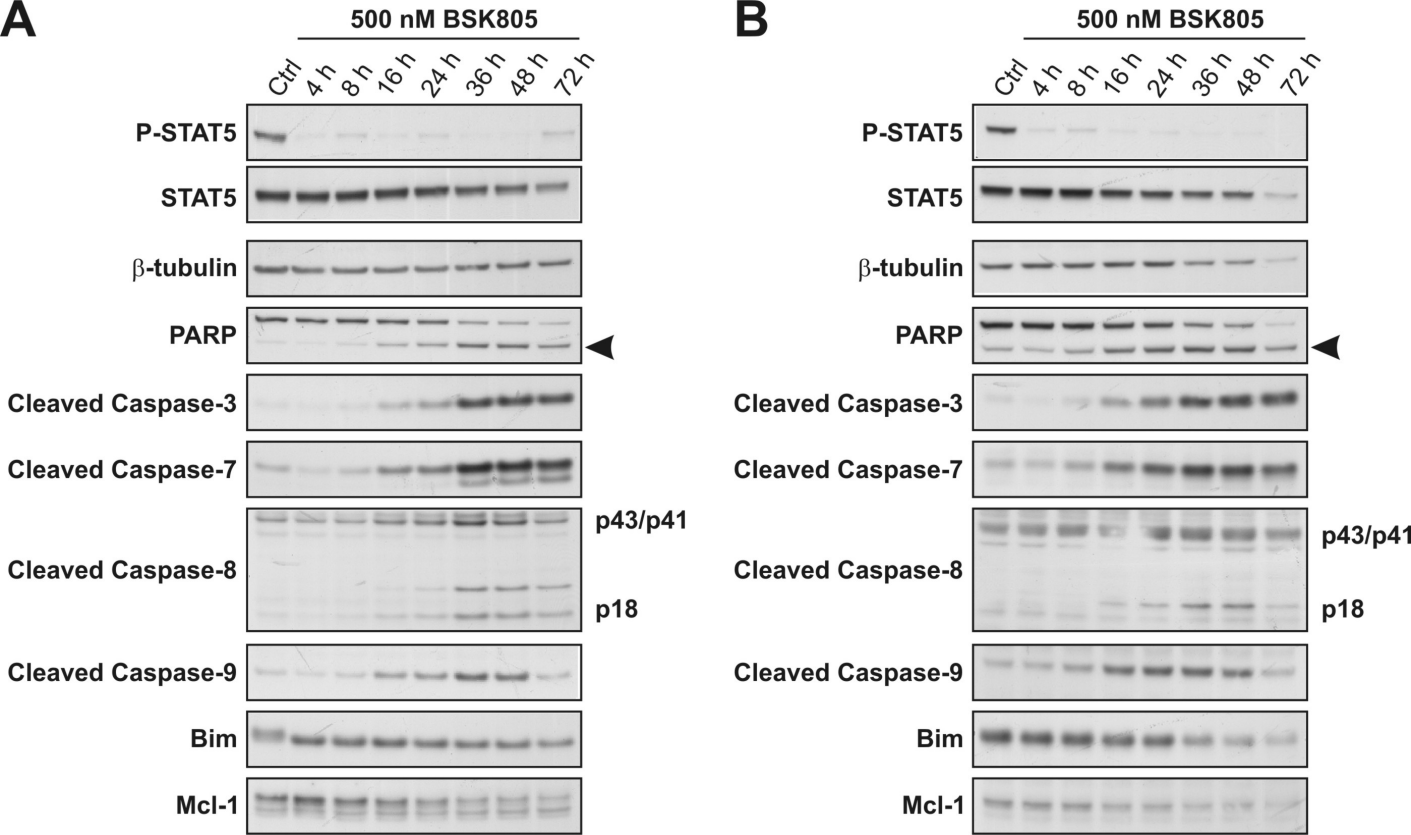
**Activation of intrinsic and extrinsic caspase cascades following JAK2 inhibition in JAK2^V617F ^mutant cells lines**. SET-2 (**A**) and MB-02 (**B**) cells were treated with 500 nM of the JAK2 inhibitor NVP-BSK805 and extracted at the indicated time points for Western blot analysis. Control (Ctrl) cells were treated with the drug vehicle DMSO for 72 hours. Induction of apoptosis markers becomes evident starting at the 16 hours time point in both cell lines. Arrowhead denotes cleaved PARP, running below the band for full-length PARP. The caspase-8 blot shows the p43/p41 cleavage intermediates and active caspase-8 fragment p18.

Apoptosis is executed by caspase cascades of the so-called intrinsic and extrinsic pathways that activate caspase 9 and 8, respectively [20]. In order to assess (i) the timing of caspase induction following JAK2 inhibition and (ii) dissect the caspase cascades triggering cell death, SET-2 and MB-02 cells lines were treated with NVP-BSK805 and extracted at different points in time. In both cell lines PARP cleavage became evident at the 16 hours time point, coinciding with the detection of cleaved caspases 9 and 8, as well as cleaved effector caspases 3 and 7 (Figure 2). These results imply that programmed cell death triggered by JAK2 inhibition in the JAK2^V617F ^mutant cell lines involves both the intrinsic and extrinsic pathways.

### Key role of Bim in JAK2 inhibitor induced apoptosis in JAK2^V617F ^cells

**Figure 3:**
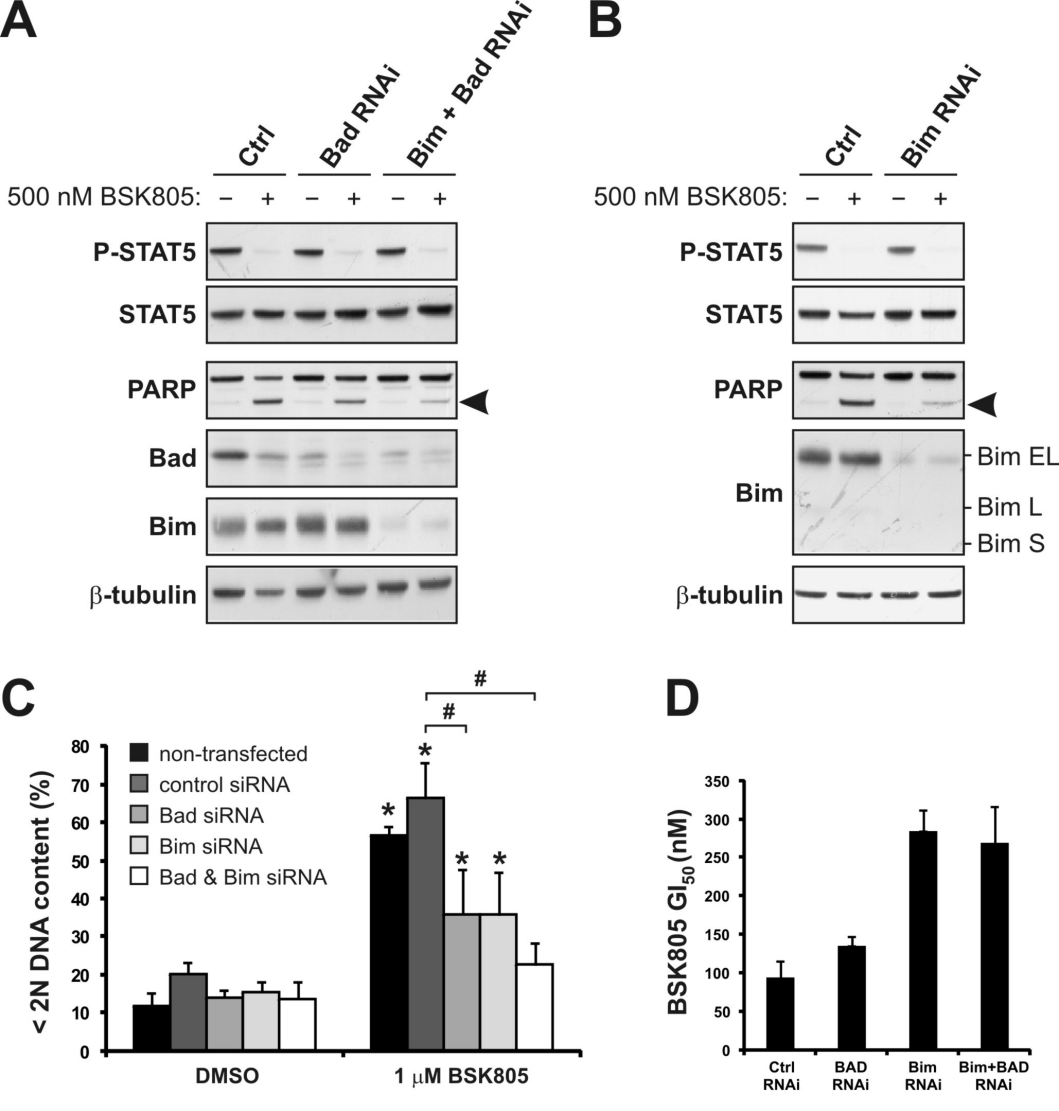
**Bad and Bim depletion in JAK2^V617F ^mutant SET-2 cells suppress NVP-BSK805-induced apoptosis**. **A**: SET-2 cells were transfected with control siRNA (Ctrl), Bad siRNA or siRNAs to deplete both Bad and Bim simultaneously. After 72 hours, cells were treated with 500 nM NVP-BSK805 or the drug vehicle DMSO for 30 hours and extracted for Western blot analysis. Arrowhead depicts cleaved PARP running below the full-length PARP protein. β-tubulin was probed for as a loading control. **B**: SET-2 cells were transfected with control siRNA (Ctrl) or Bim siRNA and analyzed as described above. The Bim Western blot depicts an extended molecular weight range to show that Bim-EL is the predominant form of the protein expressed in SET-2 cells. **C**: SET-2 cells were transfected with control, Bad or Bim siRNAs, or siRNAs to deplete both Bad and Bim simultaneously, for 48 hours and then treated with 1 μM NVP-BSK805 or the drug vehicle DMSO for 40 hours. Controls also consisted of non-transfected cells. Then, DNA content was measured by FACS using propidium iodide (PI) staining and the percentage of cells with less than 2N DNA content was determined (data represent the mean ± SD of three independent experiments). *Significantly different from respective DMSO control using *t*-test (p < 0.05). ^#^Significant difference between groups as assessed by *t*-test (p < 0.05). **D**: SET-2 cells were transfected with control, Bad or Bim siRNAs, or siRNAs to deplete both Bad and Bim simultaneously, for 24 hours and then treated with increasing concentrations of NVP-BSK805 in cell proliferation assays for 48 hours. The histogram depicts half-maximal growth-inhibitory (GI_50_) concentrations of NVP-BSK805 for each condition (data represent the mean ± SD of two independent experiments carried out in triplicate).

**Figure 4:**
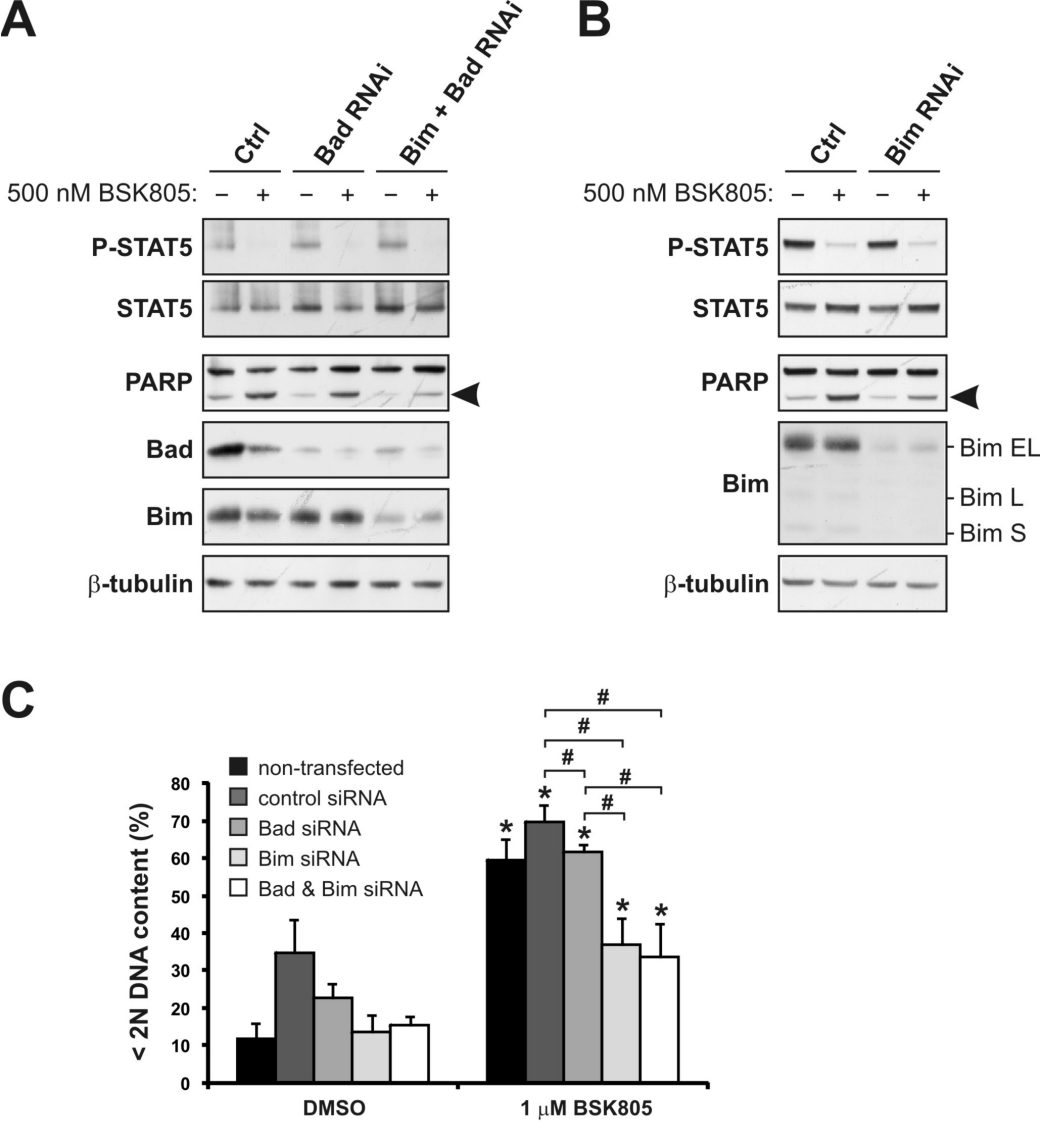
**Bim depletion in JAK2^V617F ^mutant MB-02 cells suppresses NVP-BSK805-induced apoptosis**. **A**: MB-02 cells were transfected with control siRNA (Ctrl), Bad siRNA or siRNAs to deplete both Bad and Bim simultaneously. After 72 hours, cells were treated with 500 nM NVP-BSK805 or the drug vehicle DMSO for 16 hours and extracted for Western blot analysis. Arrowhead depicts cleaved PARP running below the full-length PARP protein. β-tubulin was probed for as a loading control. **B**: MB-02 cells were transfected with control siRNA (Ctrl) or Bim siRNA and analyzed as described above. The Bim Western blot depicts an extended molecular weight range to show that Bim-EL is the predominant form of the protein expressed in MB-02 cells. **C**: MB-02 cells were transfected with control, Bad or Bim siRNAs, or siRNAs to deplete both Bad and Bim simultaneously, for 48 hours and then treated with 1 μM NVP-BSK805 or the drug vehicle DMSO for 40 hours. Controls also consisted of non-transfected cells. Then, DNA content was measured by FACS using propidium iodide (PI) staining and the percentage of cells with less than 2N DNA content was determined (data represent the mean ± SD of three independent experiments). *Significantly different from respective DMSO control using *t*-test (p < 0.05). ^#^Significant difference between groups as assessed by *t*-test (p < 0.05).

To gain more insights into the apoptotic players involved in triggering the caspases of the intrinsic pathway in JAK2^V617F ^cell lines, we tested the impact of Bad depletion on JAK2 inhibitor-induced apoptosis. Bad and Bcl-xL have previously been shown to play a role in SET-2 cell survival [10]. In agreement with these earlier reports, Bad depletion by RNAi partially suppressed apoptosis induction in SET-2 cells, as assessed by PARP cleavage and measuring the sub-G1 cell fraction by flow cytometry, following JAK2 inhibition (Figure 3A and 3C). However, in MB-02 cells Bad depletion only modestly suppressed NVP-BSK805-induced cell death (Figure 4A and 4C). Intrigued by this finding, we explored the role of Bim, another BH3-only protein, in JAK2 inhibitor-induced apoptosis. In both cell lines, Bim levels were readily detected at baseline and strongly reduced following RNAi (Figure 2, 3A and 4A). In both SET-2 and MB02 cells Bim-EL was the predominant isoform expressed (Figure 3B and 4B). Importantly, Bim-depleted SET-2 and MB-02 cells were largely resistant to cell death by NVP-BSK805 (Figure 3C and 4C). Similarly, Will *et al. *recently reported that shRNA-mediated Bim depletion suppressed apoptosis induced by JAK2 inhibition in HEL cells [21]. In SET-2 cell proliferation assays, Bim depletion resulted in a three-fold increase in the GI_50 _(half-maximal growth-inhibitory concentration) of NVP-BSK805 (Figure 3D). In agreement with a recent report [21], these findings corroborate a crucial role for Bim in the execution of cell death in JAK2^V617F ^mutant cells.

### JAK2 inhibition in JAK2^V617F ^cells modulates the post-translational modification of Bim and levels of Mcl-1

**Figure 5:**
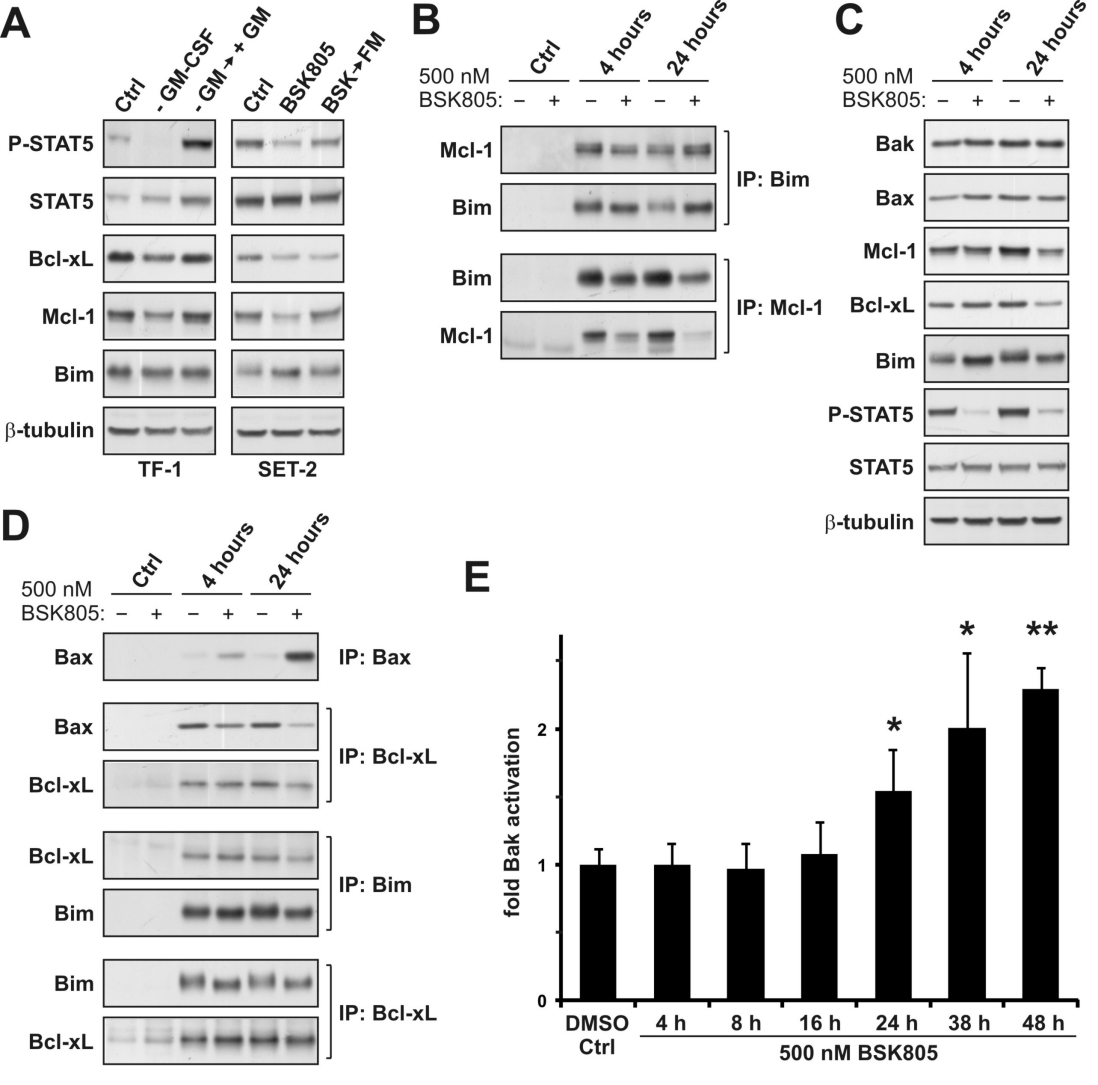
**Analysis of the regulation of anti-apoptotic and pro-apoptotic proteins in JAK2^V617F ^mutant SET-2 cells**. **A**: JAK2 wild type TF-1 cells (left panels) were starved overnight in medium containing 0.1% FCS without GM-CSF. Cells were then stimulated with 2 ng/ml GM-CSF (+GM) for 8 hours and extracted. Control TF-1 cell extracts (Ctrl) were prepared from cells growing in full medium. SET-2 cells (right panels) were treated with DMSO (Ctrl) or 500 nM NVP-BSK805 for 16 hours. Cells were either extracted or washed and released into fresh medium (FM) without NVP-BSK805 for 8 hours and then extracted. **B**: SET-2 cells were treated with DMSO or 500 nM NVP-BSK805 for 4 or 24 hours, extracted, and lysates were subjected to co-immunoprecipitation studies. To control for potential unspecific binding of the proteins of interest to beads, extracts (same total protein input as used for the immunoprecipitations) were also incubated with beads without the respective antibodies (Ctrl). **C**: Levels of soluble Bak, Bax, Mcl-1, Bcl-xL and Bim in SET-2 whole cell extracts following treatment of cells for 4 and 24 hours with DMSO or NVP-BSK805. **D**: SET-2 cell lysates from cells treated as described above in panel B were subjected to immunoprecipitation of Bax (using an antibody recognizing an amino-terminal epitope) and co-immunoprecipitation studies. Potential unspecific binding of the proteins of interest to beads was controlled (Ctrl) as described above. **E**: SET-2 cells were incubated with DMSO for 48 hours or with 500 nM NVP-BSK805 for the indicated times. Bak activation was assessed using a Bak active conformation-specific antibody by flow cytometry analysis. Results are depicted as the means of three independent experiments ± SD. *Significantly different from DMSO control using *t*-test (p < 0.05). **Significantly different from DMSO control using *t*-test (p < 0.001).

Upon incubation of JAK2^V617F ^mutant cell lines with NVP-BSK805, we noticed that Mcl-1 levels started to drop at the 16 hours time point, paralleling the activation of caspases and PARP cleavage (Figure 2). Mcl-1 is a protein with a relatively short half-life and has been shown to be dynamically regulated at the level of transcription by STAT3/STAT5 signaling [22] and at the post-translational level by phosphorylation and polyubiquitination [23,24] to signal destruction by the proteasome. To test the dynamics of Mcl-1 levels in JAK2^V617F ^cells as compared to factor-dependent cells with wild-type JAK2, we transiently blocked signaling from JAK2 to STAT5 in both contexts. Consistent with previous reports [22,25] Mcl-1 levels dropped upon starvation of TF-1 erythroleukemia cells with wild type JAK2 and recovered upon re-stimulation with GM-CSF, correlating with the changes in STAT5 phosphorylation (Figure 5A). This was very similar to the drop seen in Mcl-1 levels in JAK2^V617F^-bearing SET-2 cells after 16 hours of treatment with NVP-BSK805 and re-induction of Mcl-1 after compound washout and release of the cells into fresh medium for 8 hours (Figure 5A). Treatment of SET-2 cells with NVP-BSK805 also led to a reduction of *Mcl-1 *transcript levels, as assessed by real-time qPCR (Additional file 1). Hence, the dynamic control of Mcl-1 levels in cells with wild type JAK2 [22] appears to be maintained in JAK2^V617F ^mutant cells.

As alluded to above, Bim-EL levels were readily detectable in SET-2 and MB-02 cell lines at baseline and did not increase appreciably upon JAK2 inhibitor treatment (Figure 2A and B). This was reminiscent of the modest changes in Bim-EL levels reported in IL-3 dependent mouse pro-B FL5.12 cells following IL-3 deprivation [26]. Thus, we investigated if the association of Bim with Mcl-1 and/or Bcl-xL [15,16] would be impacted by JAK2 inhibition. Using SET-2 JAK2^V617F ^mutant cell extracts, we found that Mcl-1 co-immunoprecipitated with Bim and *vice versa *(Figure 5B). Importantly, despite a drop in total and immunoprecipitatable Mcl-1 levels in JAK2^V617F ^mutant cells treated with NVP-BSK805, the relative ratio of Bim immunoprecipitated with Mcl-1 appeared constant or even increased compared to control cell extracts, indicating enhanced association of Bim and Mcl-1 upon JAK2 inhibition (Figure 5B). Interestingly, the amounts of Mcl-1 that could be immunoprecipitated from cells treated with NVP-BSK805 were already strongly reduced at the 4 hours time point (Figure 5B), at which total levels in whole cell extracts were not yet substantially lower compared to control cells (Figure 5C). The importance of Bcl-xL in regulating survival of JAK2^V617F ^cells has already been recognized [10,27,28], hence, we also assessed its interaction with Bim [16]. Similar to the results obtained with Mcl-1, the relative amounts of Bcl-xL co-immunoprecipitated with Bim were comparable between extracts prepared from control and JAK2 inhibitor treated cells (Figure 5D), despite reduced overall levels of Bcl-xL after 24 hours of drug treatment (Figure 5C). Using an antibody that recognizes an amino-terminal epitope of human Bax, there was a pronounced increase in the amounts of detergent-soluble Bax that could be immunoprecipitated after treatment of SET-2 cells with NVP-BSK805 (Figure 5D), while the total levels of Bax were unchanged (Figure 5C). Levels of detergent-soluble Bax that could be immunoprecipitated reached a plateau by 48 hours following JAK2 inhibition (Additional file 2). These findings imply either a change of Bax conformation, or a change of multi-protein complexes containing Bax, or both upon JAK2 inhibition. In support of changes in Bim/Bcl-xL/Bax complexes following JAK2 inhibition, lower amounts of Bax co-immunoprecipitated with Bcl-xL from cells treated with NVP-BSK805 (Figure 5D). Mcl-1 was not found to co-immunoprecipitate Bax (data not shown). Importantly, besides Bax also Bak needs to be activated to trigger mitochondrial cell death [11] and Mcl-1 has been described to antagonize Bak at the mitochondrial membrane [29]. Since both Bax and Bak are expressed in SET-2 cells (Figure 5C) we investigated Bak activation following JAK2 inhibition. To this end, we carried out co-immunoprecipitation experiments to study the interaction of Bak with either Mcl-1 or Bcl-xL. Unfortunately, these analyses were confounded by unspecific binding of Bak to the beads. Thus, we assessed Bak activation by flow cytometry using a conformation-specific Bak antibody [29]. These analyses revealed significant Bak activation in SET-2 cells starting at 24 hours following JAK2 inhibition (Figure 5E).

**Figure 6:**
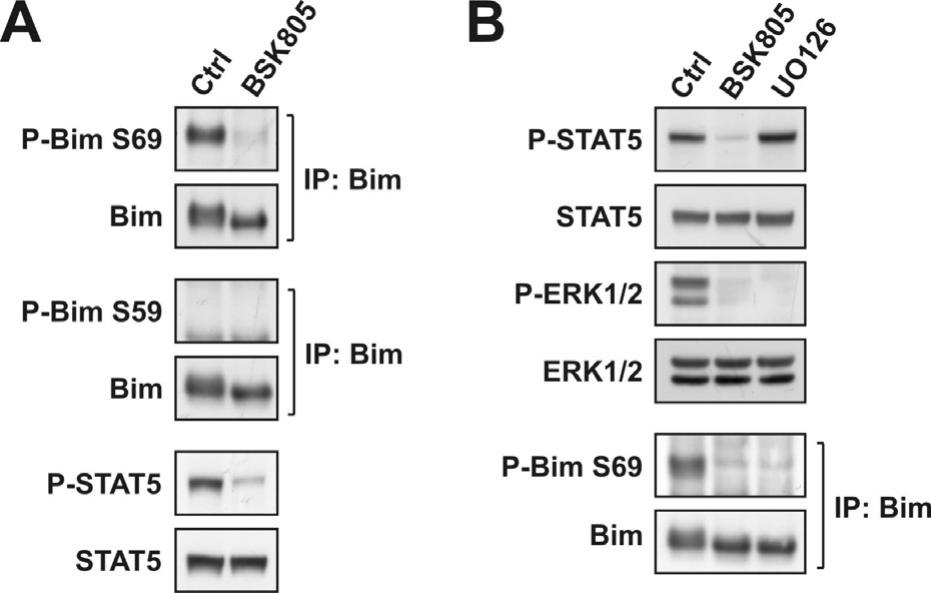
**Analysis of Bim phosphorylation in JAK2^V617F ^mutant SET-2 cells**. **A**: SET-2 cells were treated with DMSO or 500 nM NVP-BSK805 for 24 hours. Bim was immunoprecipitated and levels of Bim-EL Ser69 as well as Ser59 phosphorylation were analyzed by Western blotting. Levels of P-STAT5 and total STAT5 were probed for as controls for the drug treatment. **B**: SET-2 cells were treated with DMSO, 500 nM NVP-BSK805 or 10 μM UO126 for 4 hours. Levels of P-STAT5, STAT5, P-ERK1/2 and ERK1/2 were assessed by Western blotting. Bim was immunoprecipitated and levels of Bim-EL Ser69 phosphorylation were detected by Western blotting.

We noticed faster migration of Bim-EL in SDS-PAGE upon JAK2 inhibitor treatment (Figures 2, 3, 4, 5 and 6), indicative of changes in post-translational modification(s) [26]. Bim-EL contains a number of Ser/Thr-Pro consensus motif phosphorylation sites and phosphorylation on serine 69 by the MEK/ERK pathway was shown to regulate Bim activity/stability [26,30]. We assessed Bim Ser69 phosphorylation in SET-2 cells and found that this site was strongly modulated following JAK2 inhibition (Figure 6A), likely accounting for the changes seen in Bim-EL electrophoretic mobility, and in agreement with a recent report [21]. Phosphorylation on additional Ser/Thr-Pro sites has been reported to contribute to Bim-EL band-shifting in mouse pro-B FL5.12 cells [26]. However, we did not detect Bim Ser59 phosphorylation (Figure 6A) or Bim tyrosine phosphorylation (data not shown and [31]). In support of the MEK/ERK pathway mediating Bim phosphorylation, downstream of aberrant JAK2 signaling, treatment of SET-2 cells with the MEK inhibitor UO126 impacted Bim-EL electrophoretic mobility and Ser69 phosphorylation, comparable to that seen upon NVP-BSK805 treatment (Figure 6B).

### Mcl-1 is required for survival of JAK2^V617F ^cells

**Figure 7:**
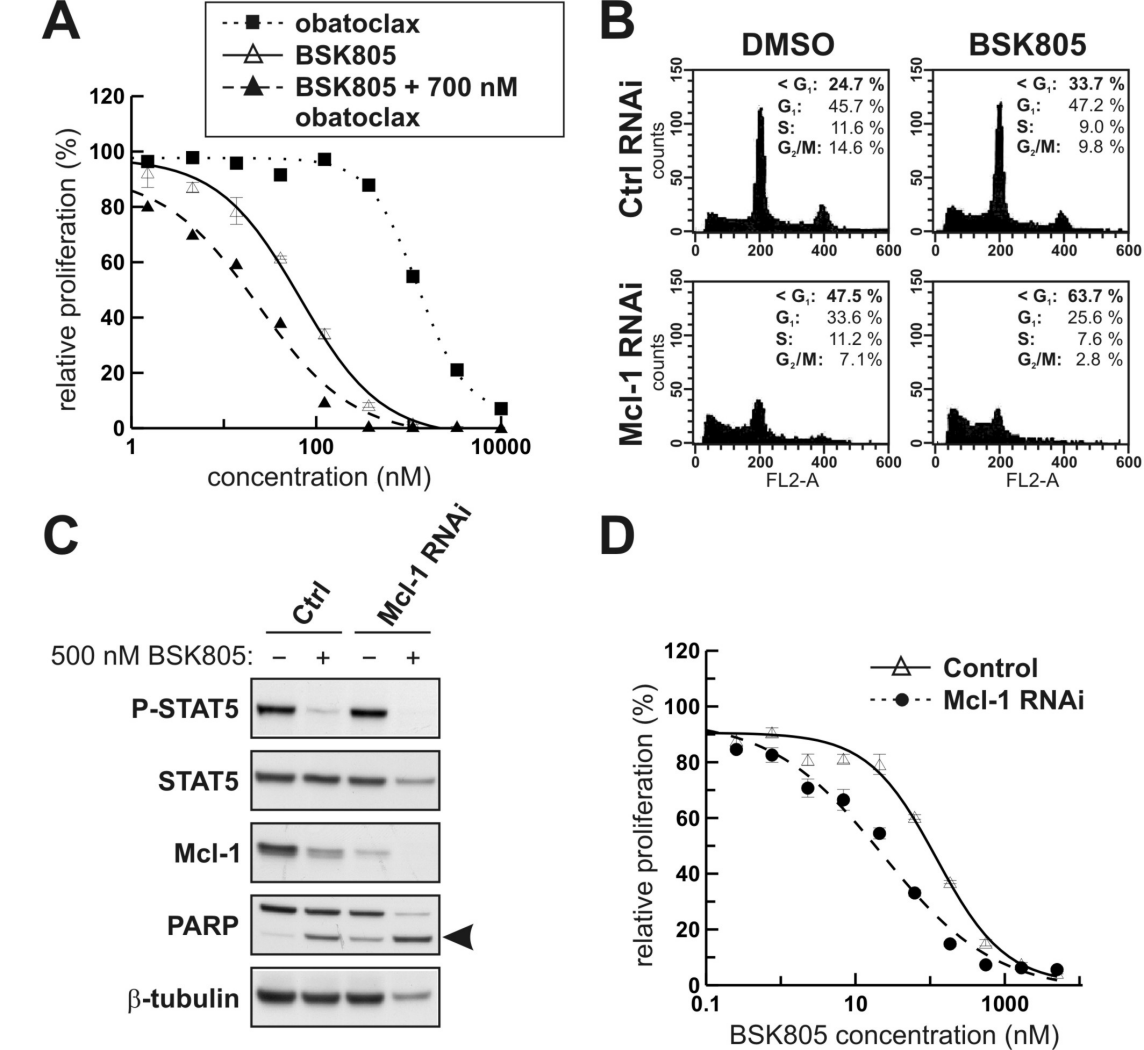
**Mcl-1 depletion in JAK2^V617F ^mutant SET-2 cells increases apoptosis and sensitizes the cells to NVP-BSK805-induced cell death**. **A**: Cell proliferation assays were carried out in SET-2 cells treated with increasing concentrations of NVP-BSK805 (empty triangles/solid line), obatoclax (filled squares/dotted line) or NVP-BSK805 in combination with a fixed obatoclax concentration of 700 nM (filled triangles/stippled line) for 72 hours. **B**: SET-2 cells were transfected with control (Ctrl) or Mcl-1 siRNAs. After 48 hours cells were treated with DMSO or 500 nM NVP-BSK805 for 24 hours. Then, DNA content was measured by FACS using propidium iodide (PI) staining. The x- and y-axes represent fluorescent channel 2-area (FL2-A) fluorescence intensity for PI staining and cell count, respectively. The percentage of cells in the respective cell cycle phases is indicated for each treatment condition. **C**: SET-2 cells were transfected with control (Ctrl) or Mcl-1 siRNAs. After 48 hours, cells were treated with 500 nM NVP-BSK805 or the drug vehicle DMSO for 24 hours and extracted for Western blot analysis. Arrowhead depicts cleaved PAPR running below the full-length PARP protein. β-tubulin was probed for as a loading control. **D**: SET-2 cells were transfected with control (Ctrl) or Mcl-1 siRNAs. After 24 hours cells were incubated for 48 hours with increasing concentrations of NVP-BSK805 to assess cell proliferation relative to the respective DMSO treated controls.

To further test the extent to which Mcl-1 plays a role in JAK2^V617F ^mutant cell survival we used approaches involving pharmacological inhibition and RNAi. Incubation of SET-2 cells with sub-optimal concentrations of the pan-Bcl-2 family protein inhibitor obatoclax [32] in cell proliferation assays lowered the GI_50 _of NVP-BSK805 by 3 to 4 fold (Figure 7A). Since obatoclax also inhibits other Bcl-2 members, besides Mcl-1, and might exhibit off-target effects [33], we expanded on these results by specifically depleting Mcl-1 using RNAi. Importantly, Mcl-1 depletion increased apoptosis in JAK2^V617F ^mutant SET-2 cells and sensitized the cells to NVP-BSK805-induced cell death as assessed by Western blot analysis and measuring the sub-G1 cell fraction by flow cytometry (Figure 7B and 7C). The latter finding was corroborated in cell proliferation assays. 24 hours after transfection of SET-2 cells with either non-targeting RNAi oligos or oligos directed towards the *Mcl-1 *transcript, cells were treated with increasing concentrations of NVP-BSK805 for 48 hours. Notably, Mcl-1 depleted SET-2 cells had an approximately 4-fold lower GI_50 _value as compared to SET-2 cells transfected with control oligos (Figure 7D). Similarly, obatoclax or Mcl-1 depletion by RNAi also strongly affected viability of MB-02 cells and sensitized them to JAK2 inhibition by NVP-BSK805 (data not shown).

## Discussion

In malignant and normal cells the balance between pro-apoptotic and anti-apoptotic signals determines cell survival. The JAK2^V617F ^mutation was identified with high frequencies in the MPNs PV, ET as well as PMF, and is thought to provide mutant progenitor cells with a proliferation and survival advantage [7]. In the present study, we have focused on assessing the roles of the pro-apoptotic protein Bim and the anti-apoptotic protein Mcl-1 in JAK2^V617F ^mutant cells. We report that Bim depletion by RNAi suppresses JAK2 inhibitor-induced apoptosis, while Mcl-1 depletion profoundly affects JAK2^V617F ^mutant cell viability and sensitizes cells to JAK2 inhibition. The BH3-only protein Bim plays an important role in hematopoietic homeostasis [34] and has been shown to be regulated by factors that activate JAK2 signaling [26,35]. Two cooperating pathways downstream of JAK2 activation have been reported to keep Bim activity in check; On one hand, PI3K/AKT signaling regulates the expression of the *Bim *gene via the forkhead transcription factor FOXO3A [36,37], whereas on the other hand, MEK/ERK signaling promotes Bim phosphorylation on Ser69 and triggers its degradation by the proteasome [30]. Furthermore, it was recently found that Bim expression in erythroblasts is suppressed by the LRF transcription factor (itself being a direct target of GATA1) in the process of erythroid maturation [38]. Mcl-1 is a member of five anti-apoptotic proteins (Bcl-2, Bcl-xL, Bcl-W, Mcl-1 and A1) that antagonize the pro-apoptotic proteins Bak and Bax [39]. Mcl-1 has a chief role in regulating the survival of hematopoietic stem cells and early hematopoietic progenitors [40]. Bcl-xL has an important role in protecting hematopoietic cells and maturing erythroid cells from cell death [41,42] and is a target gene of EpoR/JAK2 signaling [43]. Mcl-1 and Bcl-xL sequester Bak and Bax until their displacement is promoted by the action of activated BH3-only proteins to trigger subsequent mitochondrial cell death [29].

Here we show that JAK2 inhibition in JAK2^V617F ^mutant cells led to post-translational changes in Bim that affected its interaction with other Bcl-2 family members. We detected enhanced association of Bim-EL with Mcl-1 upon JAK2 inhibition, seemingly consistent with earlier findings of apoptosis induction by serum withdrawal [16]. Furthermore, there was a sharp increase in the levels of immunoprecipitable Bax following JAK2 inhibition. In various settings, Bim-EL activation also involves loss of MEK/ERK pathway-mediated Ser69 phosphorylation, whereby Bim evades proteasomal degradation [16]. Loss of Bim-EL Ser69 phosphorylation following JAK2 inhibition in the JAK2^V617F ^mutant cell lines analyzed in this study likely plays a role in Bim activation, in agreement with a recent study by Will *et al. *[21]. However, Will *et al. *reported that Bim protein levels were up-regulated in JAK2^V617F ^mutant cells following JAK2 inhibition [21], which we did not see in our analyses. These differences might be attributable to different experimental settings. In fact, using factor-independent Ba/F3 pro-B cells stably expressing EpoR and JAK2^V617F ^we also detected low basal levels of Bim-EL and a marked up-regulation upon JAK2 inhibition (data not shown), as found by Will *et al*. However, Ba/F3 cells do not represent the hematopoietic lineage in which the JAK2^V617F ^mutation arises and regulation of Bim activity may be cell lineage-specific [26]. Taken together, our findings imply that Bim is in a latent complex with the Bcl-2 family pro-survival proteins Mcl-1 and Bcl-xL in viable JAK2^V617F ^mutant cells. Both Mcl-1 and Bcl-xL govern survival of JAK2^V617F ^mutant cells by keeping Bax and Bak in check. In turn, JAK2 inhibition is postulated to affect Bim complexes such that Mcl-1 and Bcl-xL are neutralized. This is proposed to drop anti-apoptotic activity in JAK2^V617F ^mutant cells below a critical threshold, unleashing Bak and Bax to drive mitochondrial cell death. Upon inhibition of JAK2/STAT signaling the expression of Bcl-xL [44] and Mcl-1 [25,45] is suppressed, along with subsequent reduction of Bcl-xL and Mcl-1 protein levels, thereby contributing to the loss of pro-survival activity. Hence, as in CML [46-48] and FLT-3 mutant [49] AML cells, Bim is also emerging as a central cell death driver in JAK2^V617F ^mutant cells ([21], and this report).

Polycythemia vera patients with high JAK2^V617F ^mutant allele burden were described to have increased levels of Bcl-2 as well as Bcl-xL, and the Bcl-2/Bcl-W/Bcl-xL inhibitor ABT-737 was shown to preferentially inhibit proliferation and induce mitochondrial depolarization in JAK2^V617F ^mutant erythroblasts as compared to those from healthy subjects [9]. However, at the level of the individual MPN patient, Zeuner *et al.* did not detect a strict correlation between Bcl-2 or Bcl-xL expression and drug resistance, indicating that response to therapy may be determined by additional underlying anti-apoptosis mechanisms. Our findings suggest that combinations of JAK2 inhibitors with Bcl-2 family antagonists that also tackle Mcl-1, besides Bcl-xL [21,50], merit further preclinical evaluation of the therapeutic potential for the treatment of cMPNs. Importantly, partial inhibition of Mcl-1 may be sufficient to sensitize cells to JAK2 inhibition. This could be important in order to minimize the impact on normal cells, such as e.g. on B and T lymphocytes, in which Mcl-1 plays a key role, as revealed by conditional knock-out studies [51]. Furthermore, it will be of particular interest to explore if combinations of JAK2 inhibitors with Bcl-2 family antagonists result in enhanced killing of the MPN mutant clone. Thus, follow-up experiments in suitable preclinical MPN animal models [52-54] would be important for proof of concept *in vivo *and to support the translation of potentially promising therapeutic modalities into the clinical setting. Encouragingly, clinical assessment of JAK inhibitors in MPN patients is underway [55], as well as intense drug discovery and development efforts to identify Mcl-1 antagonists [32,56].

## Conclusions

Bim and Mcl-1 were found to have opposing roles in regulating JAK2^V617F ^cell survival. JAK2 inhibition in JAK2^V617F ^mutant cells led to loss of Bim-EL Ser69 phosphorylation, with concomitant enhanced sequestration of the Bcl-2 family proteins Mcl-1 and Bcl-xL. Consistent with a key role of Bim in regulating apoptosis in JAK2^V617F ^mutant cells, depletion of the BH3-only protein by RNAi markedly suppressed JAK2 inhibitor-induced cell death. *Vice versa*, RNAi-mediated Mcl-1 depletion sensitized JAK2^V617F ^mutant cells to JAK2 inhibition. Thus, further preclinical assessment of combinations of JAK2 inhibitors with Bcl-2 family antagonists in models of cMPNs is warranted and antagonizing Mcl-1, besides Bcl-xL, should be an integral part of such strategies.

## Competing interests

All authors are full-time employees of Novartis Pharma AG.

## Authors' contributions

JR and ZQ carried out the majority of the studies in JAK2^V617F ^mutant cell lines, participated in the design of experiments and helped draft parts of the manuscript. RA performed experiments in the cellular models and carried out Western blot analysis of Bim phosphorylation. DAG performed analyses of pro- and anti-apoptotic proteins. TR conceived the study, participated in the design of experiments and drafted the manuscript. All authors read and approved the final manuscript.

## Pre-publication history

The pre-publication history for this paper can be accessed here:

http://www.biomedcentral.com/1471-2407/11/24/prepub

## Supplementary Material

Additional file 1 **Supplementary Figure S1 - Reduction of *Mcl-1 *transcript levels following JAK2 inhibition by NVP-BSK805 in JAK2^V617F ^mutant SET-2 cells**. SET-2 cells were treated for 4 and 8 hours with 500 nM NVP-BSK805. Control cells were treated with the drug vehicle DMSO for 4 hours. Total RNA was isolated and *Mcl-1 *transcript levels were determined in triplicate by real-time quantitative PCR. *Mcl-1 *mRNA levels were normalized to *GAPDH *mRNA levels in the respective samples and means ± SD were expressed as fold change compared to the DMSO treated sample. Similar results were obtained in two independent experiments. Click here for file

Additional file 2 **Time course of Bax activation following JAK2 inhibition by NVP-BSK805 in JAK2^V617F ^mutant cell lines**. SET-2 (**A**) and MB-02 (**B**) cells were treated with 500 nM of the JAK2 inhibitor NVP-BSK805 and extracted at the indicated time points for immunoprecipitation and Western blot analysis. Control (Ctrl) cells were treated with the drug vehicle DMSO for 48 hours. Cells were extracted in lysis buffer containing 1% CHAPS and Bax was immunoprecipitated using an antibody that recognizes the amino-terminal epitope that is exposed in the active conformation of Bax. Levels of immunoprecipitatable Bax at the different time points following JAK2 inhibition were detected by Western blotting. Western blot analysis was also used to assess levels of Bax, PARP (cleaved PARP is depicted by arrowheads) and β-tubulin in whole cell extracts. Results are representative of two independent experiments. Click here for file
