# Supplementary figures and images for: Bim and Mcl-1 exert key roles in regulating JAK2V617F cell survival
Source: BMC Cancer. 2011 Jan 19;11:24. doi: 10.1186/1471-2407-11-24 (PMC3037340; doi:10.1186/1471-2407-11-24)

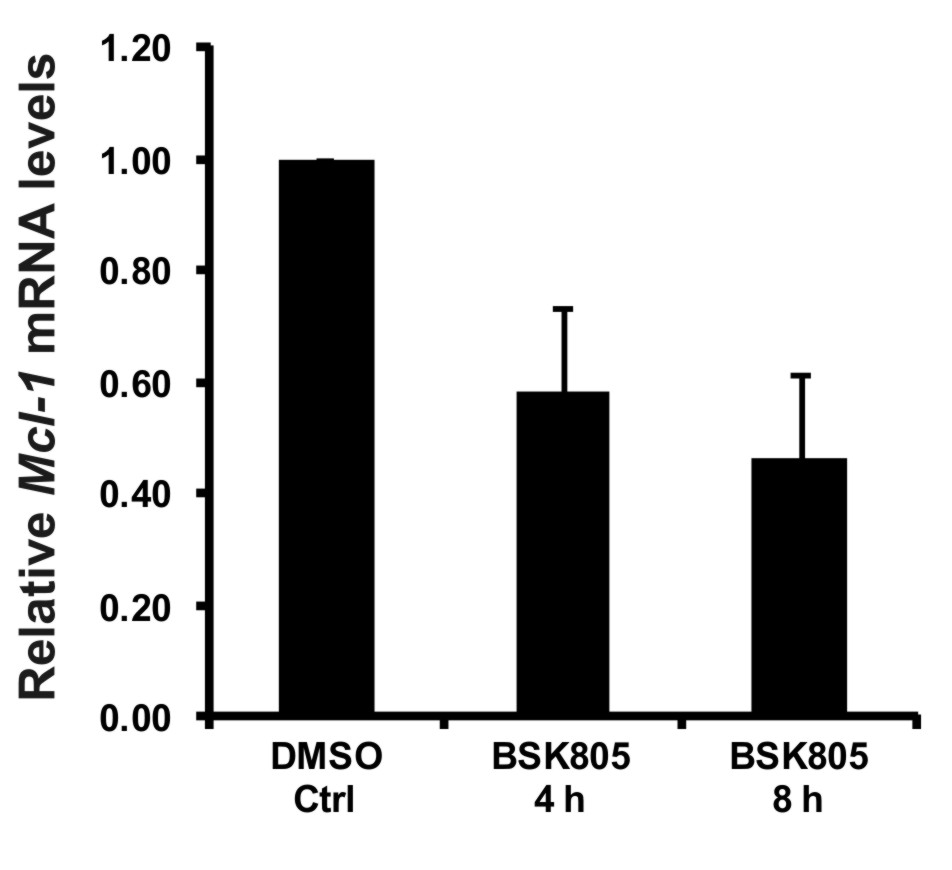

Supplement: Additional file 1 — Supplementary Figure S1 - Reduction of Mcl-1 transcript levels following JAK2 inhibition by NVP-BSK805 in JAK2V617F mutant SET-2 cells. SET-2 cells were treated for 4 and 8 hours with 500 nM NVP-BSK805. Control cells were treated with the drug vehicle DMSO for 4 hours. Total RNA was isolated and Mcl-1 transcript levels were determined in triplicate by real-time quantitative PCR. Mcl-1 mRNA levels were normalized to GAPDH mRNA levels in the respective samples and means ± SD were expressed as fold change compared to the DMSO treated sample. Similar results were obtained in two independent experiments. [file 1471-2407-11-24-S1.JPEG]

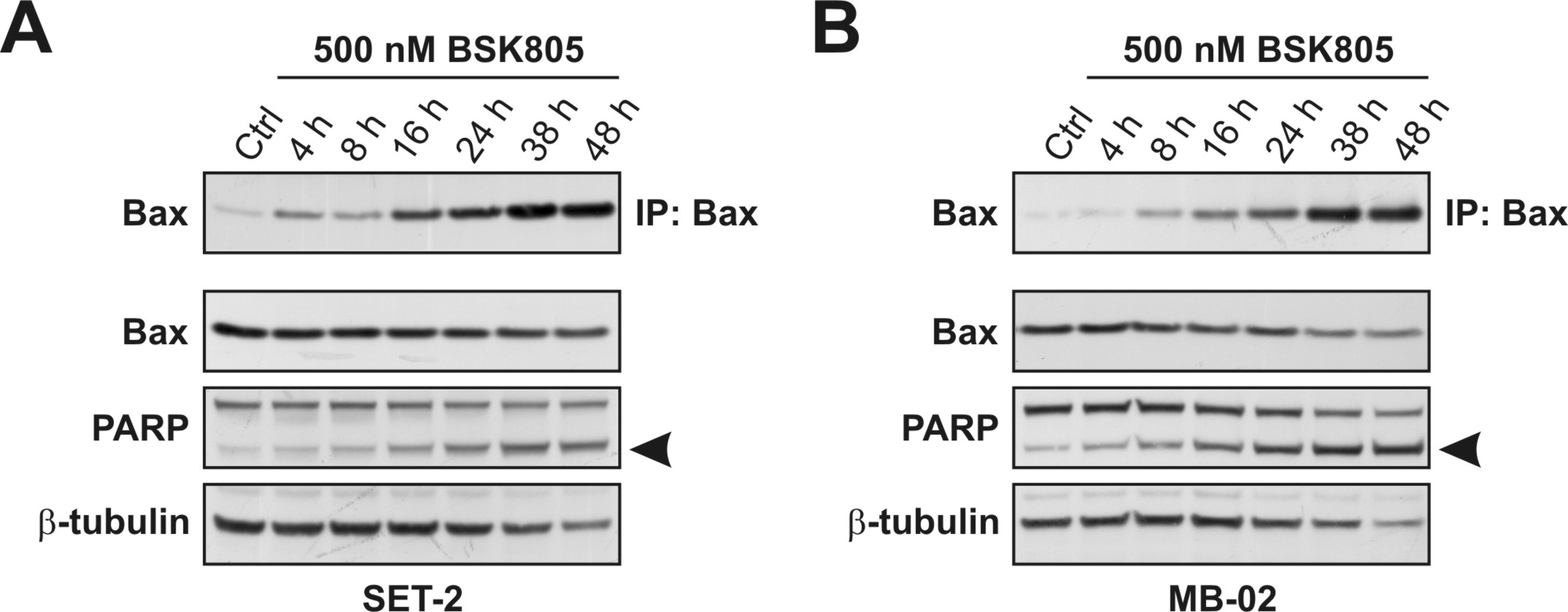

Supplement: Additional file 2 — Time course of Bax activation following JAK2 inhibition by NVP-BSK805 in JAK2V617F mutant cell lines. SET-2 (A) and MB-02 (B) cells were treated with 500 nM of the JAK2 inhibitor NVP-BSK805 and extracted at the indicated time points for immunoprecipitation and Western blot analysis. Control (Ctrl) cells were treated with the drug vehicle DMSO for 48 hours. Cells were extracted in lysis buffer containing 1% CHAPS and Bax was immunoprecipitated using an antibody that recognizes the amino-terminal epitope that is exposed in the active conformation of Bax. Levels of immunoprecipitatable Bax at the different time points following JAK2 inhibition were detected by Western blotting. Western blot analysis was also used to assess levels of Bax, PARP (cleaved PARP is depicted by arrowheads) and β-tubulin in whole cell extracts. Results are representative of two independent experiments. [file 1471-2407-11-24-S2.JPEG]
